# Development of an antibacterial polypropylene/polyurethane composite membrane for invisible orthodontics application

**DOI:** 10.3389/fbioe.2023.1233398

**Published:** 2023-07-07

**Authors:** Feng Yang, Chenyi Wu, Yuanzhang Jiang, Lin Tan, Rui Shu

**Affiliations:** ^1^ State Key Laboratory of Oral Diseases, Department of Pediatric Dentistry, West China School of Stomatology, Sichuan University, Chengdu, China; ^2^ State Key Laboratory of Polymer Materials Engineering, College of Biomass Science and Engineering, Sichuan University, Chengdu, China; ^3^ Research Center for Fiber Science and Engineering Technology, Yibin Institute of Industrial Technology, Sichuan University, Yibin, China

**Keywords:** membrane, antibacterial, antibiofilm, antifouling, invisible orthodontics

## Abstract

In virtue of the advantages, such as aesthetics, designability, convenient removal, and comfortable experience, invisible orthodontics (IO) have been widely recognized and accepted by the public. However, most of the membranes currently used for IO only meet the requirement of shape retention. Other vital functions, like antibacterial and antifouling activities, are neglected. Herein, antibacterial composite membranes (ACMs) containing polypropylene (PP), thermoplastic polyurethane (TPU) and poly (hexamethylene guanidine) hydrochloride-sodium stearate (PHMG-SS) were facilely manufactured through the hot-pressing membrane forming technology. ACMs were conferred with favorable transparency (∼70% in the visible light range) and excellent antibacterial ability. Experiment results demonstrated that bactericidal rates of ACMs against *Staphylococcus aureus*, *Escherichia coli* and *Streptococcus mutans* were larger than 99.99%. Noticeably, the amount of protein adhered on the surface of ACMs was only 28.1 μg/cm^2^, showing ideal antifouling performance. Collectively, the mutifunctional ACMs in the study are expected to be prominent alternatives for existing IO.

## 1 Introduction

In recent decades, the health and whitening of teeth has received widespread attention (Sims et al., 2020). Orthodontics is becoming extensively popular as the most efficient and simplest treatment method for teeth restoration. Orthodontics can not only prevent teeth from oral problems (including periodontitis, caries, tooth loss, etc.), but also can achieve the effect of teeth whitening and improve the self-confidence of patients (Burden, 2007; Chaturvedula et al., 2021). At present, compared with traditional orthodontics, invisible orthodontics (IO) has gradually become the mainstream with its advantages of aesthetics comfort and ease of use. However, the antibacterial ability of the membrane used in traditional IO is insufficient or even missing, which makes it easy for microorganisms to accumulate, adhere and proliferate in oral environment (Schlafer et al., 2012). When wearing IO, the flow rate of saliva between IO and the dental mucosa will slow down, seriously affecting the self-cleaning of teeth and oral tissues, which will further lead to the growth of bacteria (Kuramitsu et al., 2007; Kolenbrander et al., 2010). Therefore, the development of functional membrane with antibacterial and antifouling properties for the application of IO is of great significance for the prevention of bacterial adhesion and colonization, thereby promoting the oral health and safety during orthodontic treatment.

Among the several parameters affecting the clinical performance of IO, the membranes used for the manufacture of the IO play a crucial role (Bichu et al., 2023). According to literature reports, poly (methyl methacrylate) (PMMA), poly (ethylene terephthalate-1,4-cyclohexanedimethanol) (PETG), polypropylene (PP), thermoplastic polyurethane (TPU), polycarbonate (PC) and ethylene vinyl acetate (EVA) have been applied in IO fabrication (Gardner et al., 2003; Kwon et al., 2008; Zhang et al., 2011; Lombardo et al., 2017). However, the IO membranes prepared through conventional process usually have no antibacterial and low antifouling abilities. Therefore, various strategies have been adopted to prevent the growth and colonization of bacteria between IO and teeth, such as adding antibacterial agents, antibacterial coatings and constructing self-cleaning surfaces (Milionis et al., 2020; Zhang et al., 2020; Worreth et al., 2021). Xie et al. presented a strategy for modifying orthodontic devices, which took quaternary ammonium modified gold nanoclusters as an antibiotic reagent to prevent bacterial contamination and biofilm formation (Xie et al., 2020). Cheng reported an interpenetrating polymer network (IPN) structured antibacterial layer that was prepared on dental base materials containing quaternary ammonium salt monomers (HMDQAs), polyurethane dimethacrylate oligomers (BIHs) and functionalized SiO_2_ nanoparticles, and this coating exhibited good transparency, excellent antibacterial activity and low cytotoxicity (Cheng et al., 2019). However, antibacterial coatings usually involve unstable fastness and influence on the physical properties of the substrate material, and the coating agents were often dissolved in toxic organic solvents, which were not environmentally friendly, and may further damage the surface structure of the coating substrate, and other related issues.

Polypropylene (PP) is considered an important polymer material due to its ease of processing, cheap price, high chemical resistance and good mechanical properties, which has been widely used in medical equipment, building materials, transportation and other fields (Zhao et al., 2021). As the demand for healthy life increasing, materials such as polymer membranes with antibacterial activity have attracted much attention in the medical and health fields, and their excellent antibacterial properties can alleviate or even solve the problem of infection and disease transmission (Huang et al., 2007). At present, the simplest processing method that can impart antibacterial capacity to PP is through the melt blending with antibacterial agents. Chen et al. fabricated antibacterial PP by incorporating silver nanoparticles, when the amount of silver nanoparticles reached 8wt%, 100% of *E. coli* and more than 99% of *S. aureus* were killed (Chen et al., 2020). Besides, dodecyl mercaptan functionalized silver nanoparticles were introduced into PP membrane by melt-blending, and the inhibition rates of the antibacterial PP membrane against *E. coli S. aureus* reached 100% and 84.6%, respectively (Cao et al., 2018). However, due to the non-polar nature of PP, it often faces the uneven dispersion when combined with inorganic powder, and also undergoes the weaknesses, such as poor wear resistance, low weather resistance, and difficult functionalization. Therefore, it is often compound with other polymer chips. Thermoplastic polyurethane (TPU) commonly exhibits excellent impact strength, flexibility and excellent wear resistance. It can improve the mechanical properties of the composites by blending with PP (Bakare et al., 2008). Therefore, by designing organic antibacterial PP/TPU composite membranes (ACMs) with low price and excellent processing performance, endowing them with excellent mechanical properties (such as breaking strength, tensile deformation, wear resistance, etc.), which can be applied for manufacturing invisible braces and other biomedical devices.

In present study, PP, TPU and organic PHMG-SS antibacterial agent were employed to prepare ACMs through the facile hot-pressing technology. The obtained ACMs were characterized in terms of mechanical, morphological and surface features. The results indicated that the ACMs exhibited favorable transparency, excellent antibacterial performance, protein adhesion resistance, and ideal cytotoxicity, and it is expected to be applied as the membrane substrate for IO.

## 2 Materials and methods

### 2.1 Materials

Polypropylene (PP) (S2040) was purchased from Shanghai Secco Petrochemical Co., Ltd., thermoplastic polyurethane (TPU) (Elastollan^®^, Mw∼220000) was purchased from BASF (China) Co., Ltd., and poly (hexamethylene guanidine) hydrochloride-sodium stearate (PHMG-SS) antibacterial compound was prepared with reference to our previous literature (Zhang et al., 2021), potassium dihydrogen phosphate, sodium chloride, potassium chloride, agar powder, yeast extract powder and other reagents were from Chengdu Kelong Chemical Co., Ltd., and tryptone was from Beijing Aoboxing Biotechnology Co., Ltd.; *Staphylococcus aureus* (*S. aureus*, ATCC6538), *Escherichia coli* (*E. coli*, ATCC8739) were provided by the Functional Fiber Research Laboratory of Sichuan University, and *Streptococcus mutans* (*S. mutans*) was provided by West China Hospital of Sichuan University.

### 2.2 Preparation of ACMs

PP, TPU and PHMG-SS were dried for 12 h to remove moisture. PP and TPU with a total mass of 10 g (different proportions) were mixed for membrane formation through hot-pressing, and PHMG-SS was added during the membrane formation process with two ratios, including 0.5% and 1.0% relative to total mass. The parameter settings are as follows: lamination temperature ∼210°C, lamination including preheating 3 min, precompression 3 min, and pressurization 5 min, exhaust ∼2 times, and the applied pressure was 15 bar/cm^2^. After the pressing, the mould was withdrawn, and the membranes were collected after cooling naturally at room temperature. Specifically, ACMs were denoted according to the compositions, and named as PxTy, where x, y represent the mass fraction of PP and TPU, for example, when the ratio of PP and TPU was 4:6, the amount of PHMG-SS added were 0, 0.5% and 1%, which were named P4T6, P4T6 (0.5%) and P4T6 (1%), accordingly.

### 2.3 Characterization of ACMs

The optical transparency of the ACMs was characterized by an UV-visible-near-infrared spectrometer (PerkinElmer 1,050, United States), and the surface wettability was measured by a contact angle goniometer (HARKE-SPCAX 1, China). The mechanical properties of ACMs were tested by a strength tester (YM06E, China). The sample width was 5 mm, the clamp distance was 10 mm, and the tensile speed was 10 mm/min. Each sample was tested five times, and the average values of breaking strength and elongation at break were recorded. The thermal stability of the ACMs was tested by thermogravimetry analysis (PerkinElmer TGA 8000, United States). The test temperature was ranging from 30°C to 800°C, and the heating rate was 10°C/min. The surface potential of the ACMs was tested by a solid/membrane surface ZETA potentiometer (Anton Paar, SurPASS2, Austria). Field emission scanning electron microscopy (SEM, SU3500, Japan) was used to characterize the surface and cross-sectional morphologies of the ACMs. Atomic Force Microscope (AFM, Dimension ICON, Germany) was used to observe the surface roughness of the ACMs, and NanoScope Analysis 1.8 was used for analysis.

### 2.4 Protein adsorption performance test

All membranes (1 cm × 1 cm) were pre-wetted in Phosphate Buffered Saline (PBS, pH = 7.4) buffer, and then placed in bovine serum albumin (BSA) solution at room temperature for 12 h. After being gently washed with PBS for 5 times, the membranes were placed in 1 mL of PBS and shaken at room temperature for 2 h to elute the protein adsorbed on the membranes surface. Then, the protein elution solution from each membrane was added to the 96-well plate containing the bicinchoninic acid (BCA) reagent and then left at room temperature for 30 min. The amount of adsorbed protein was determined by testing the absorbance at 562 nm by using a microplate reader (Multiskan FC, Thermo, United States). Each sample was tested five times.

### 2.5 Determination of minimum inhibitory concentration

The antibacterial test refers to the shaking method (Zhou et al., 2022). The bacteria were cultured in liquid culture medium at 37°C to logarithmic-phase, and the bacterial suspension was diluted to 10^5^∼10^6^ CFU/mL with sterile PBS. Then 1, 5, and 10 mg of the ACMs were mixed with 1 mL of the diluted bacterial solution, and the blank control was the bacterial solution without ACMs. Afterwards, the mixtures were cultivated at 37°C for 12 h, and the bacteria solution were diluted to an appropriate concentration by 10-fold dilution method and spread onto the agar plates for the final colony counting.

### 2.6 Antibacterial kinetic test

Similar to above process, the bacteria were cultured to logarithmic-phase, then diluted to 10^5^∼10^6^ CFU/mL with PBS, 10 mg of P4T6 (1%) membranes were added to bacterial solution for co-culture, and antibacterial tests were carried out with different contact periods (1 h, 2 h, 4 h, 8 h). 100 μL of the bacterial solution was diluted and spread onto the agar plates for the final colony counting and the number of recorded colonies was applied to calculate the antibacterial ratios.

### 2.7 Live and dead bacteria staining

The bacteria were cultured to logarithmic-phase, the ACMs were added to the bacterial solution for incubation at 37°C for 24 h, and the free-standing bacteria on the surface of the membrane were gently removed with PBS, and then the membranes were stored in the dark. SYTO 9 and PI were used to double-stain the membranes under dark conditions and the staining of bacteria were observed by confocal laser scanning microscope (CLSM) (Liu et al., 2022). Furthermore, the deposited bacteria after washing on the membranes were immobilized through 2.5% glutaraldehyde aqueous solution for 4 h, then dehydrated in ethanol gradient solutions (25%, 50%, 75%, 100%), and each gradient ethanol was placed for 15 min to achieve the dehydrated bacteria. Finally, the adhesion and structural of bacteria were observed by SEM.

### 2.8 Antibacterial stability test

In order to simulate the application environment of the human oral cavity, the ACMs were soaked in artificial saliva for different periods (6, 12 h) under 37°C, and then washed with distilled water, dried at room temperature. After that, the antibacterial effect was again investigated according to the above antibacterial test process.

### 2.9 Antibiofilm property (crystal violet staining)

The ACMs were completely immersed in the suspensions containing *S. aureus*, *E. coli* and *S. mutans* (2*10^8^ CFU/mL, 1 mL) for 48 h to form biofilms on the surface. After the membranes were cleaned with PBS to remove the free-standing bacteria, 1 mL of methanol was added to fix them for 15 min in the dark. After that, the methanol was sucked off and evaporated naturally, then the ACMs were incubated with 0.1% crystal violet solution for 15 min, and the unbound crystal violet was washed away by PBS. Then, 1 mL of 95% ethanol was added to dissolve the crystal violet, and the absorbance of the dissolved crystal violet solution was measured at 490 nm using a microplate reader. Each sample was tested five times.

### 2.10 Cytotoxicity

According to the GB/T16886.5-2003 standard, mouse skeletal muscle cells L929 and cell counting kit-8 assay (CCK-8) were used to detect the cytotoxicity of the ACMs. Firstly, the ACMs were soaked in the DMEM medium containing 10% FBS and 1% penicillin streptomycin at 37°C for 12 h to obtain the ACMs extracts. Simultaneously, L929 cells were seeded on a 96-well plate at 5,000 cells/well, and then cultured in a CO_2_ incubator at 37°C for 12 h to allow the cells to adhere to the bottom. Then, the DMEM medium was replaced with the ACMs extracts, and the 96-well plates were placed in the incubator for further 3 days. After each day, 200 μL of CCK-8 was added into each well for incubation in the dark for 2 h. Finally, the absorbance values were recorded at a wavelength of 490 nm, and the formula for calculating cell viability was as follows:

$$Cell\;Viability\;\left(\%\right)=\left(A_{W}-A_{B}\right)/\left(A_{N}-A_{B}\right)\times 100$$

Among them, *A_W_*, *A_N_*, *A_B_* are the absorbance value of the sample, negative control and blank control at wavelength of 490 nm, respectively. Each sample was tested five times.

## 3 Results and discussion

### 3.1 Characterization of ACMs

**FIGURE 1:**
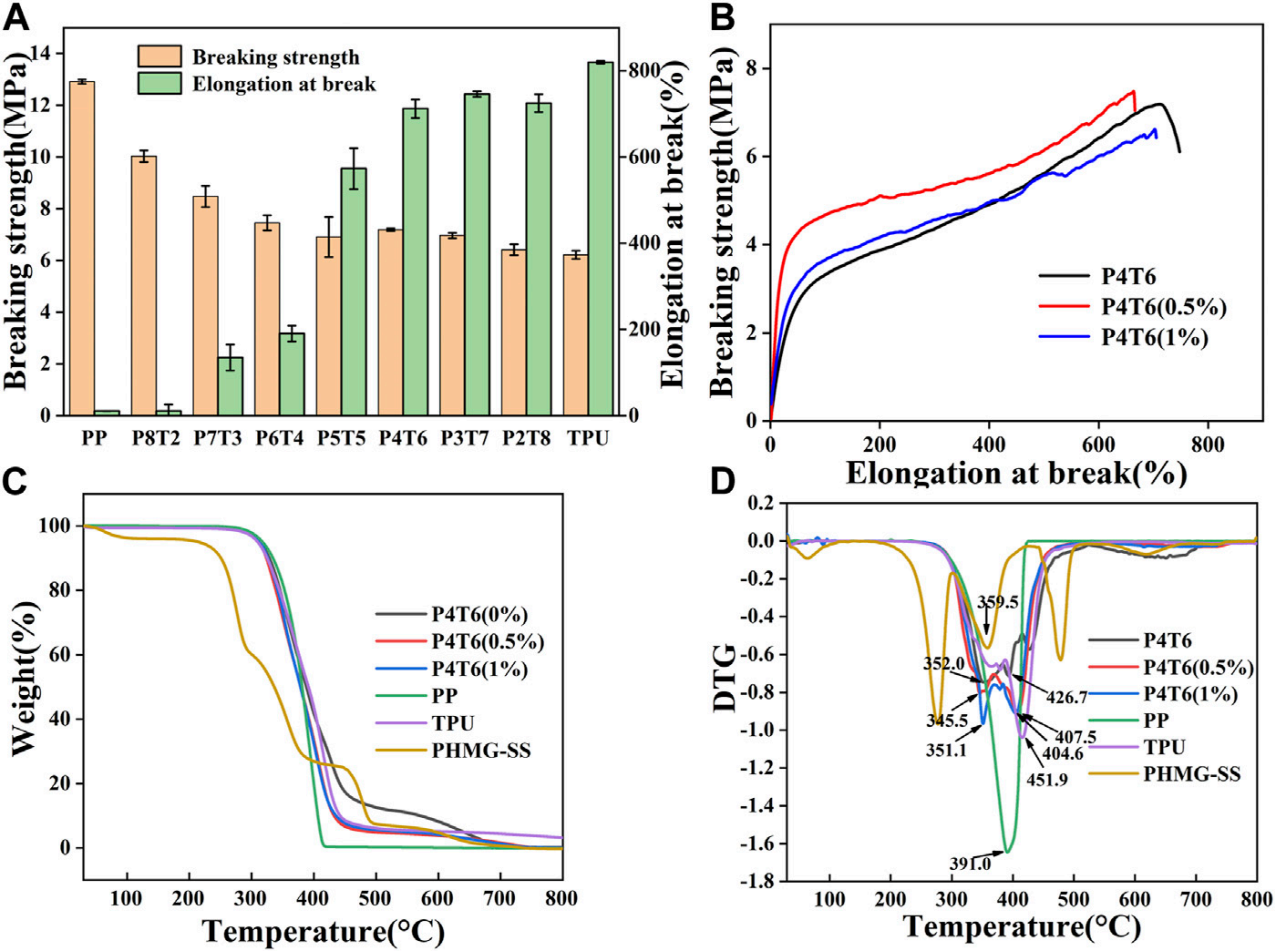
**(A)** Mechanical properties of composite membranes with different ratios; **(B)** Mechanical properties of the ACMs; TG **(C)** and DTG **(D)** curves of ACMs.

Due to the water absorption feature of TPU, if the moisture content is too high, a large number of bubbles will be generated during hot-pressing, which will negatively affect the membrane-forming process. Therefore, PP and TPU have been dried before pressing, and the contents of moisture are shown in Supplementary Table S1. The mechanical properties of composite membranes are summarized in Figure 1A, and it can be found that when P4T6 exhibits the relatively best mechanical properties in terms of tensile strain and strength which are 360.5% and 7.1 MPa, respectively. After adding PHMG-SS, the mechanical properties only slightly decrease (Figure 1B). The reason is that the addition of PHMG-SS may cause stress concentration, which lead to the decline of mechanical properties.

Figures 1C, D show the thermal analysis results of the ACMs. The maximum thermal decomposition temperature of the pure composite membrane exhibits two peaks, which are 352.0°C and 462.7°C, respectively, indicating that the membrane contains two substances (referring to PP and TPU). After the addition of PHMG-SS, both the decomposition temperature and the thermal stability decreased. The possible reason is that the SS components can accelerate the degradation of PP/TPU (Richert et al., 2019), but the ACMs still maintain good thermal stability which can satisfy the thermal sterilization process.

### 3.2 Surface topography analysis

**FIGURE 2:**
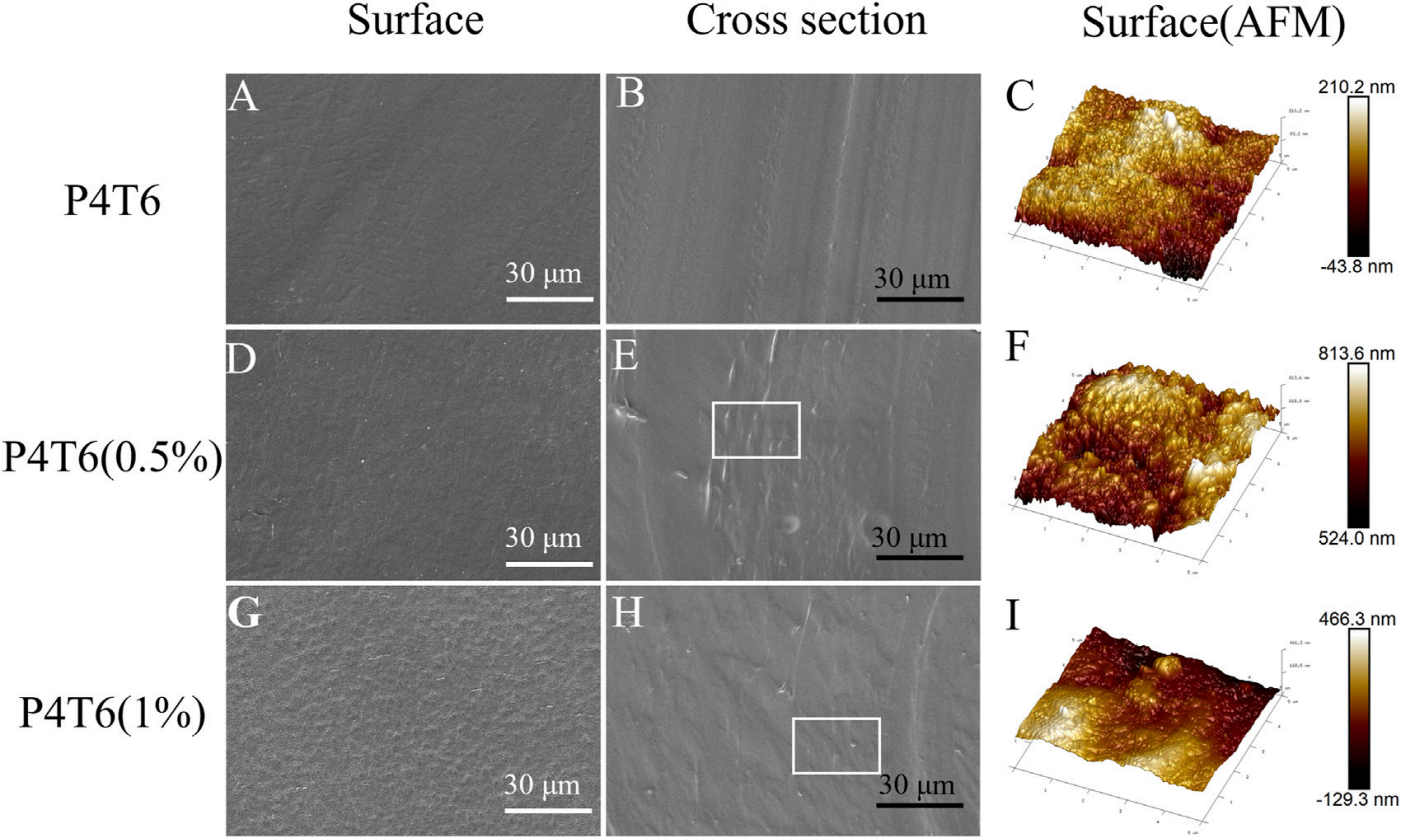
SEM images of the surfaces and cross-section of P4T6 **(A,B)**, P4T6 (0.5%) **(D,E)**, and P4T6 (1%) **(G,H)**, and AFM images of the surfaces of P4T6 **(C)**, P4T6 (0.5%) **(F)**, and P4T6 (1%) **(I)**.

From the SEM images of the surface and cross section of ACMs shown in Figure 2; Supplementary Figure S4, the surface of P4T6 is smooth, and the cross-section does not show obvious phase separation, indicating that the melt processing compatibility between TPU and PP. According to the measurement, the thickness of P4T6, P4T6 (0.5%), and P4T6 (1%) are 98.3, 98.0 and 93.9 μm, respectively, which indicate that PHMG-SS has no significant effect on film thickness. As the content of PHMG-SS increases, the surface of the membranes gradually becomes rougher, because PHMG-SS influences the interaction between PP and TPU macromolecular chains, and a small amount of unmelted PHMG-SS may also increase the surface roughness of the ACMs. This result was further verified by AFM test. According to the images of AFM (Figures 2C, F, I), the Rq values of P4T6, P4T6 (0.5%), and P4T6 (1%) are 27.6, 32.1 and 40.2 nm, respectively, where Rq represents the root mean square roughness. The lower the Rq value, the smoother the surface of the membranes.

### 3.3 Surface wettability and antifouling performance

**FIGURE 3:**
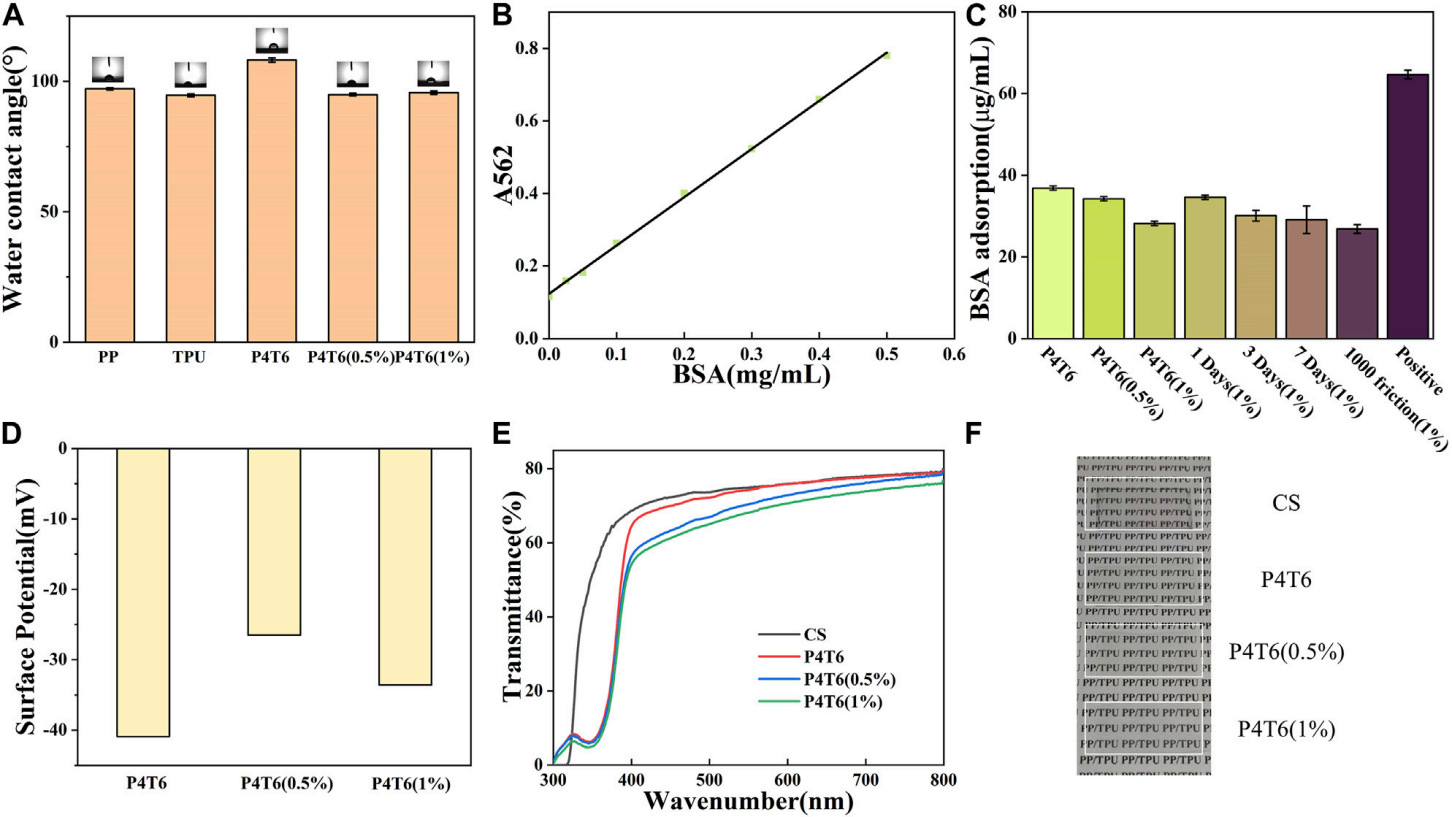
**(A)** Water contact angle of different membranes; **(B)** BSA standard curve; **(C)** BSA adsorption amounts; Surface potentials **(D)**, UV **(E)** and visible light **(F)** transmittance of the ACMs.

The water contact angle (WCA) is an important indicator of surface wettability. As shown in Figure 3A, the WCAs of pure PP and TPU are 97.1° and 94.6°, while that of P4T6 is 108.1°. Compared with pure PP and TPU, the WCA of their blender increase due to the increased roughness. When PHMG-SS was incorporated into the system, the hydrophobicity decreased, which can be attributed to the presence of hydrophilic guanidine groups in PHMG-SS. However, the WCA value (95.7°) of P4T6 (1%) is slightly higher than that (94.9°) of P4T6 (0.5%). The possible reason is the influence from the geometrical surface roughness and hydrophilic guanidine groups.

The static protein adsorption performance can reflect the antifouling performance of the ACMs. The BSA standard curve is shown in Figure 3B, and the fitting curve is y=1.33x+1.23 . By substituting the absorbance of the BSA eluate of the ACMs into the standard curve, the BSA adsorption amounts of various membranes are summarized in Figure 3C. The protein adsorption capacity of the positive control (cellulose acetate membrane) is 64.6 mg/cm^2^, while the protein adsorption capacities of other membranes are lower than 40 μg/cm^2^, indicating good antifouling performance of the prepared membranes. The surface potentials are shown in Figure 3D, because both ACMs and protein are negatively charged, thus there exists electrostatic repulsion between them, so it shows good resistance to protein adhesion. The BSA adsorption amounts of P4T6, P4T6 (0.5%), and P4T6 (1%) are 36.8, 34.2 and 28.1 μg/cm^2^, respectively. As the content of PHMG-SS increases, the antifouling performance also gradually increases, because the guanidine groups on PHMG-SS can combine with water molecules and form a hydration layer (Regev et al., 2022). Besides, the hydrophilic property of the membrane surface is also an important factor affecting protein adsorption, and the introduction of PHMG-SS leads to an increase in hydrophilicity (Yuan et al., 2021). In summary, the reduction of protein adsorption is attributed to the PHMG-SS. Considering the application scenario, friction resistance test was conducted for ACMs. As shown in Supplementary Figure S5, after 1,000 times of friction, the weight of the three samples still remains above 99%, showing excellent wear resistance. In addition, owning to long-term application in the oral environment, the antifouling properties of materials are highly susceptible to be affected. Hence, the antifouling stability of the resulting membranes was investigated. As shown in Figure 3C, there is no significant change in the amount of BSA adsorbed by the membranes after all the treatments, including the soaking in in artificial saliva for 1, 3, 7 Days, and after 1,000 times of friction, indicating their good stability.

The optical transparency of IO directly affects its aesthetics. In this study, the optical transparency of the ACMs was characterized by UV-visible spectroscopy. Considering the aesthetics of IO, we fabricated the ACMs with low roughness and good transparency, due to a relatively smoother surface can improve its transparency (Zhong et al., 2021)). Figure 3E shows the transmittance spectra of P4T6, P4T6 (0.5%) and P4T6 (1%). In fact, the transmittance curves of the three samples are very close at 300–800 nm, and reached 60% at 500 nm, which was similar to commercial sample (CS). In addition, as shown in Figure 3F, the words on the paper can be clearly seen through the ACMs. Therefore, the ACMs exhibit good optical transparency close to the commercial product, which can meet the transparency requirements of IO.

### 3.4 Characterization of antibacterial properties

**FIGURE 4:**
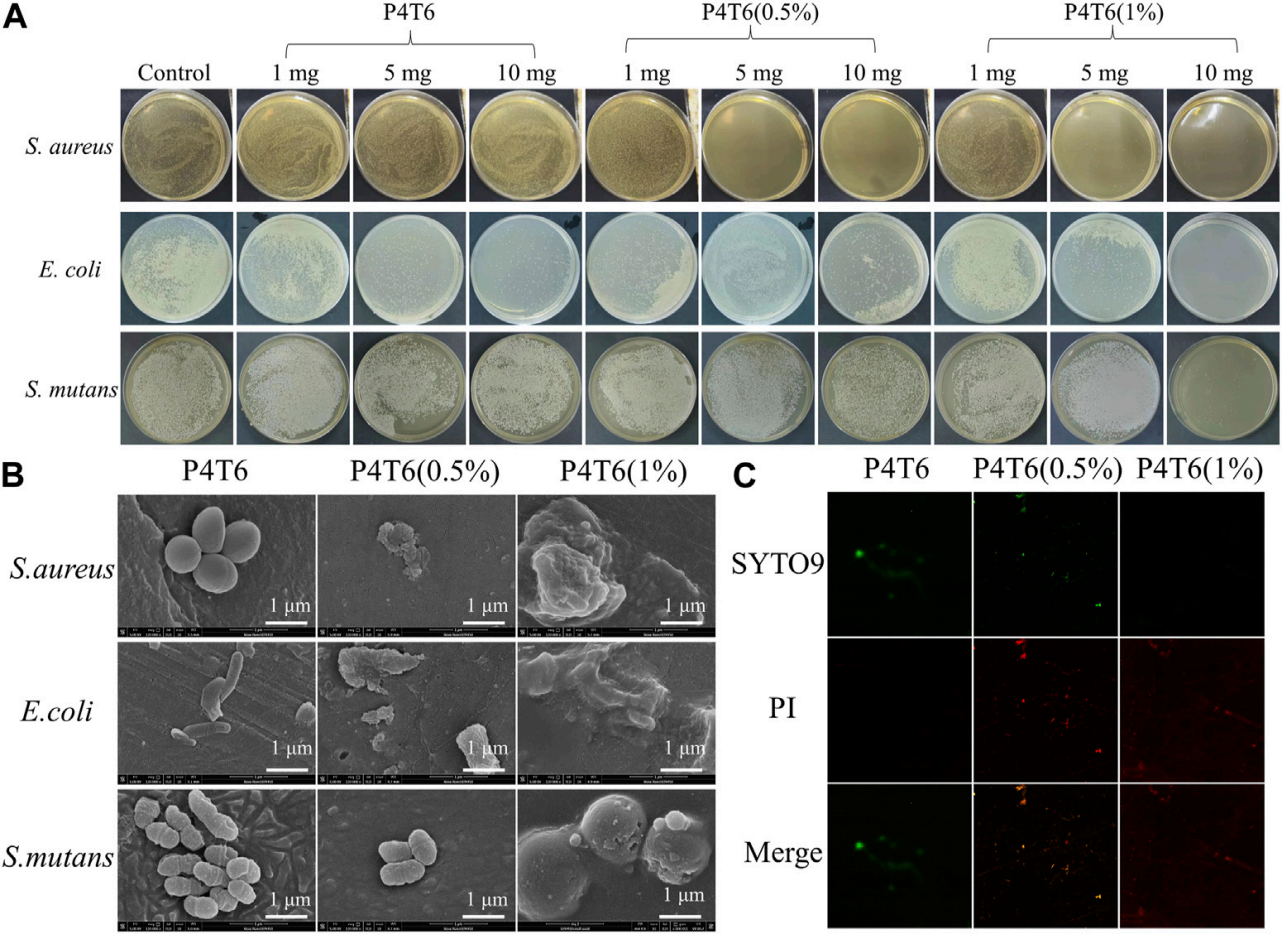
**(A)** Minimum inhibitory concentration of ACMs investigation; SEM **(B)** and CLSM **(C)** images of bacteria on ACMs.

As a common broad-spectrum antibacterial agent, PHMG shows good antibacterial properties against *S. aureus* and *E. coli* (Zheng et al., 2020). *S. mutans*, a bacterium that widely exits in environment, will secrete acidic substances to corrode tooth enamel, which is one of the main cariogenic bacteria. Therefore, the antibacterial properties of the membranes were investigated with *S. aureus*, *E. coli*, and *S. mutans*. The antibacterial results of P4T6 (0.5%) and P4T6 (1%) against the above three bacteria are shown in Figure 4A, and the MIC values of P4T6 (1%) against *S. aureus*, *E. coli* and *S. mutans* (∼10^6^ CFU/mL) are all less than 10 mg (reference to GB/T20944.3-2008). Both ACMs exhibit bactericidal rates of 99.999% for *E. coli* and *S. aureus*, and a bactericidal rate of 99.997% for *S. mutans*, indicating their extraordinary antibacterial properties. Overall, the antibacterial effect of ACMs against *E. coli* is better than *S. aureus* and *S. mutans*, which may be attributed to the thicker peptidoglycan layer of the cell walls of Gram-positive bacteria (*S. aureus* and *S. mutans*) than that of Gram-negative bacteria (*E. coli*), and this layer can protect them from antibacterial agents (Yang et al., 2021).

The adhesion of *S. aureus*, *E. coli* and *S. mutans* on ACMs were observed by SEM. As shown in Figure 4B, the morphologies of *S. aureus*, *E. coli* and *S. mutans* on the surface of P4T6 are intact and independent, contrastively, three types of bacterial cells on the surface of P4T6 (1%) membrane rupture, partially collapse and fuse together, indicating that P4T6 (1%) exhibits a destructive effect on the bacterial cells, which finally cause bacterial death as PHMG containing positively charged and hydrophobic alkyl groups can act on negatively charged bacterial membranes, leading to bacterial lysis and death (Jiang et al., 2018). Furthermore, with increasing the content of PHMG-SS, the number of bacteria adhered to the surface of the membrane also decrease, demonstrating that the ability of ACMs on preventing bacterial adhesion. Also, the surface electronegativity of ACMs may benefit the reduction of antibacterial adhesion owing to the electrostatic repulsion interaction.

In addition, the antibacterial activity of the ACMs was analyzed by fluorescence staining experiment. Syto 9 can label all bacteria with intact and damaged membranes, while PI can only label damaged bacterial cells with high permeability (Yang et al., 2020; Wang et al., 2023), and the introduction of PI will cause the decrease in the fluorescence intensity of Syto 9. Therefore, when dyed by the mixed solution of Syto 9 and PI, the bacteria with complete membrane structure will show green fluorescence, and the bacteria with damaged membrane structure will show red fluorescence. Images of bacteria after fluorescent staining are shown in Figure 4C. In the control group (P4T6), live *S. mutans* (green fluorescence) can be found presented in green color. With increasing the content of PHMG-SS, the number of live bacteria decreased and the number of dead bacteria presented in red color increased, and there is almost no green fluorescence on P4T6 (1%) membrane, which means that *S. mutans* were all killed. Meanwhile, with the introduction of PHMG-SS, the fluorescence intensity decreases, which is consistent with the above antibacterial adhesion observed by SEM. Moreover, the photos of fluorescent stained *S. aureus* and *E. coli* treated by P4T6 (1%) are shown in Supplementary Figure S6.

### 3.5 Antibacterial kinetics and antibiofilm performance

**FIGURE 5:**
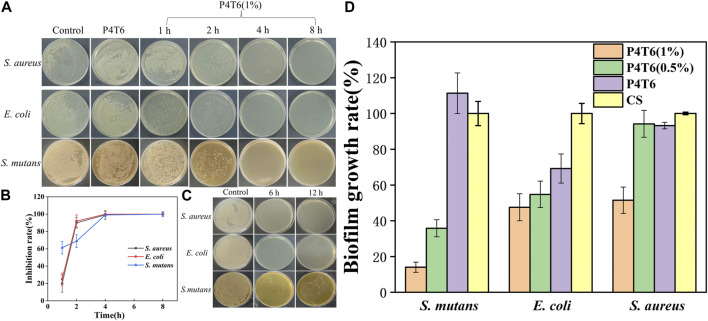
**(A)** Antibacterial kinetics of P4T6 (1%); **(B)** Sterilization curves with different contact periods; **(C)** Bactericidal ability after artificial saliva treatment (6, 12 h); **(D)** Biofilm growth ratios after crystal violet staining.

The sterilization rates of ACMs against *S. aureus*, *E. coli* and *S. mutans* at different contact periods are shown in Figure 5A, P4T6 exhibits no antibacterial effect, but as expected, P4T6 (1%) membrane exhibits an antibacterial ability which is positively correlated with contact time (Figure 5B). After 4 h of contact, >99% bacterial were killed. In addition, after the treatment with artificial saliva for 6 and 12 h, the antibacterial ability of P4T6 (1%) still remains above 90% against three types of bacteria (Figure 5C), indicating the antibacterial property of P4T6 (1%) is relatively stable which may benefit the practical application needs of orthodontic patients and can ensure the oral health.

The antibiofilm performance of the ACMs are summarized in Figure 5D; Supplementary Figure S7. Taking the commercial sample as a comparison, with increasing the content of PHMG-SS, the biofilm growth ratios are significantly reduced, and the biofilm growth ratios of the three bacteria after the treatment by P4T6 (1%) (14.05% for *S. mutans*, 47.55% for *E. coli*, 51.47% for *S. aureus*) are much lower than those by the control group. On the one hand, the guanidine group on the surface of ACMs kill bacteria and inhibit the formation of biofilms; on the other hand, the hydrophobic surface also prevents the adhesion of bacteria. In brief, P4T6 (1%) can effectively inhibit the formation of biofilms on its surface.

### 3.6 Cytotoxicity

**FIGURE 6:**
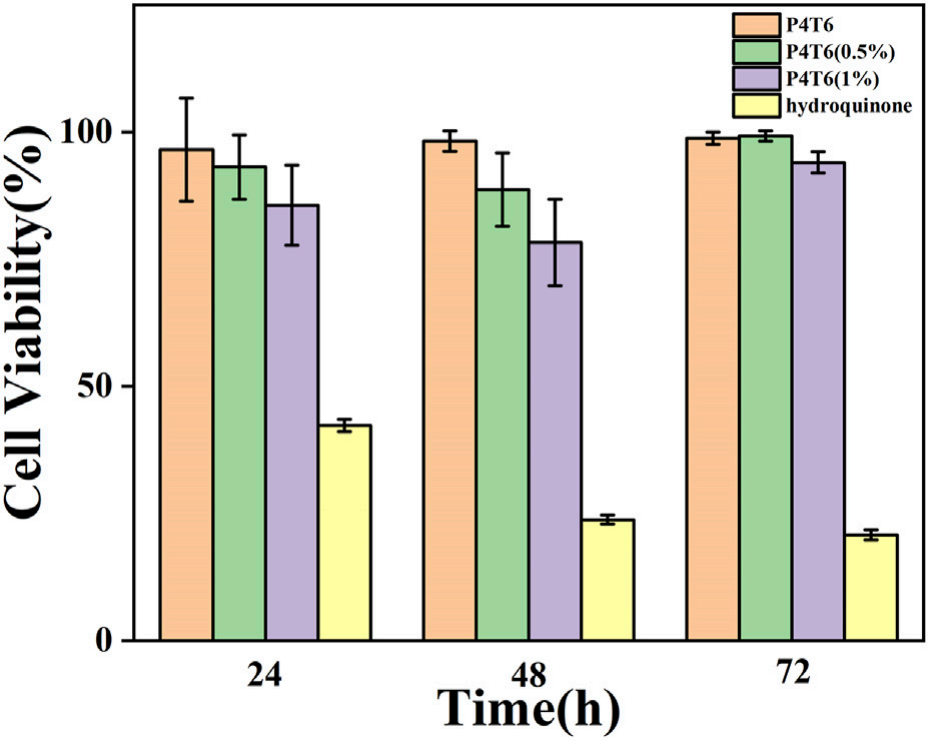
Cytotoxicity test results at P4T6, P4T6 (0.5%), P4T6 (1%) and hydroquinone (positive control).

In addition, in order to further evaluate the safety of the ACMs on the human body, cytotoxicity experiments are conducted using the extracts of P4T6, P4T6 (0.5%), P4T6 (1%) and hydroquinone, respectively. The experimental results (Figure 6) show that the cell viability of all ACMs groups are above 80%, and the relative growth ratio of the P4T6 (1%) group is the lowest, but still higher than 75%. According to the standard toxicity class (GB/T16886.5-2003), their cytotoxicity belongs to class 1, which means ACMs meet the biosafety requirements.

## 4 Conclusion

In this study, ACMs with favorable transparency and excellent antibacterial performance were prepared by one-step hot-pressing membrane forming technology. The bactericidal rates against *S. aureus*, *E. coil* and *S. mutans* can reach 99.999%, 99.999%, and 99.997%, respectively. In addition, study have also revealed that ACMs had great antifouling property, which can effectively prevent the adhesion and growth of bacteria, and excellent biocompatibility (the cell viability was above 75%). Therefore, the ACMs designed and prepared in this study can be a potential alternative product to commercial IO.

## Data Availability

The original contributions presented in the study are included in the article/Supplementary Material, further inquiries can be directed to the corresponding author.

## References

[B1] Bakare I. O. Pavithran C. Okieimen F. E. Pillai C. K. S. (2008). Synthesis and characterization of rubber-seed-oil-based polyurethanes. J. Appl. Polym. Sci. 109 (5), 3292–3301. 10.1002/app.28391

[B2] Bichu Y. M. Alwafi A. Liu X. Andrews J. Ludwig B. Bichu A. Y. (2023). Advances in orthodontic clear aligner materials. Bioact. Mater 22, 384–403. 10.1016/j.bioactmat.2022.10.006 36311049 PMC9588987

[B3] Burden D. J. (2007). Oral health-related benefits of orthodontic treatment. Semin. Orthod. 13 (2), 76–80. 10.1053/j.sodo.2007.03.002

[B4] Cao G. Lin H. Kannan P. Wang C. Zhong Y. Huang Y. (2018). Enhanced antibacterial and food simulant activities of silver nanoparticles/polypropylene nanocomposite films. Langmuir 34 (48), 14537–14545. 10.1021/acs.langmuir.8b03061 30398355

[B5] Chaturvedula B. B. Muthukrishnan A. Bhuvaraghan A. Sandler J. Thiruvenkatachari B. (2021). Dens invaginatus: A review and orthodontic implications. Br. Dent. J. 230 (6), 345–350. 10.1038/s41415-021-2721-9 33772187

[B6] Chen J. Fan L. Yang C. Wang S. Zhang M. Xu J. (2020). Facile synthesis of Ag nanoparticles-loaded chitosan antibacterial nanocomposite and its application in polypropylene. Int. J. Biol. Macromol. 161, 1286–1295. 10.1016/j.ijbiomac.2020.07.151 32693127

[B7] Cheng Q. Guo X. Zou J. Shi X. Ding S. Shi Z. (2019). A photo-crosslinked hybrid interpenetrating polymer network (IPN) for antibacterial coatings on denture base materials. New J. Chem. 43 (42), 16647–16655. 10.1039/c9nj02609f

[B8] Gardner G. D. Dunn W. J. Taloumis L. (2003). Wear comparison of thermoplastic materials used for orthodontic retainers. Am. J. Orthod. Dentofac. Orthop. 124 (3), 294–297. 10.1016/s0889-5406(03)00502-x 12970663

[B9] Huang J. Murata H. Koepsel R. R. Russell A. J. Matyjaszewski K. (2007). Antibacterial polypropylene via surface-initiated atom transfer radical polymerization. Biomacromolecules 8 (5), 1396–1399. 10.1021/bm061236j 17417906

[B10] Jiang S. Wang W. Ding Y. Yu Q. Yao L. (2018). Preparation and characterization of antibacterial microporous membranes fabricated by poly(AMS-co-DMAEMA) grafted polypropylene via melt-stretching method. Chin. Chem. Lett. 29 (3), 390–394. 10.1016/j.cclet.2018.01.006

[B11] Kolenbrander P. E. Palmer R. J. Jr. Periasamy S. Jakubovics N. S. (2010). Oral multispecies biofilm development and the key role of cell-cell distance. Nat. Rev. Microbiol. 8 (7), 471–480. 10.1038/nrmicro2381 20514044

[B12] Kuramitsu H. K. He X. Lux R. Anderson M. H. Shi W. (2007). Interspecies interactions within oral microbial communities. Microbiol. Mol. Biol. Rev. 71 (4), 653–670. 10.1128/mmbr.00024-07 18063722 PMC2168648

[B13] Kwon J. S. Lee Y. K. Lim B. S. Lim Y. K. (2008). Force delivery properties of thermoplastic orthodontic materials. Am. J. Orthod. Dentofac. Orthop. 133 (2), 228–234. 10.1016/j.ajodo.2006.03.034 18249289

[B14] Liu X. Guo C. Zhuang K. Chen W. Zhang M. Dai Y. (2022). A recyclable and light-triggered nanofibrous membrane against the emerging fungal pathogen Candida auris. PLoS Pathog. 18 (5), e1010534. 10.1371/journal.ppat.1010534 35613180 PMC9173615

[B15] Lombardo L. Martines E. Mazzanti V. Arreghini A. Mollica F. Siciliani G. (2017). Stress relaxation properties of four orthodontic aligner materials: A 24-hour *in vitro* study. Angle Orthod. 87 (1), 11–18. 10.2319/113015-813.1 27314603 PMC8388588

[B16] Milionis A. Tripathy A. Donati M. Sharma C. S. Pan F. Maniura-Weber K. (2020). Water-Based scalable methods for self-cleaning antibacterial ZnO-nanostructured surfaces. Ind. Eng. Chem. Res. 59 (32), 14323–14333. 10.1021/acs.iecr.0c01998 32831473 PMC7434054

[B17] Regev C. Jiang Z. Kasher R. Miller Y. (2022). Critical surface density of zwitterionic polymer chains affect antifouling properties. Appl. Surf. Sci. 596, 153652. 10.1016/j.apsusc.2022.153652

[B18] Richert A. Olewnik-Kruszkowska E. Adamska E. Tarach I. (2019). Enzymatic degradation of bacteriostatic polylactide composites. Int. Biodeterior. Biodegrad. 142, 103–108. 10.1016/j.ibiod.2019.04.010

[B19] Schlafer S. Meyer R. L. Sutherland D. S. Stadler B. (2012). Effect of osteopontin on the initial adhesion of dental bacteria. J. Nat. Prod. 75 (12), 2108–2112. 10.1021/np300514z 23167781

[B20] Sims K. R. Jr. Maceren J. P. Liu Y. Rocha G. R. Koo H. Benoit D. S. W. (2020). Dual antibacterial drug-loaded nanoparticles synergistically improve treatment of Streptococcus mutans biofilms. Acta Biomater. 115, 418–431. 10.1016/j.actbio.2020.08.032 32853808 PMC7530141

[B21] Wang X. Wang H. Cheng J. Li H. Wu X. Zhang D. (2023). Initiative ROS generation of Cu-doped ZIF-8 for excellent antibacterial performance. Chem. Eng. J. 466, 143201. 10.1016/j.cej.2023.143201

[B22] Worreth S. Bieger V. Rohr N. Astasov‐Frauenhoffer M. Töpper T. Osmani B. (2021). Cinnamaldehyde as antimicrobial in cellulose‐based dental appliances. J. Appl. Microbiol. 132 (2), 1018–1024. 10.1111/jam.15283 34480822 PMC9292871

[B23] Xie Y. Zhang M. Zhang W. Liu X. Zheng W. Jiang X. (2020). Gold nanoclusters-coated orthodontic devices can inhibit the formation of Streptococcus mutans biofilm. ACS Biomater. Sci. Eng. 6 (2), 1239–1246. 10.1021/acsbiomaterials.9b01647 33464842

[B24] Yang T. Li N. Wang X. Zhai J. Hu B. Chen M. (2020). Dual functional AgNPs-M13 phage composite serves as antibacterial film and sensing probe for monitoring the corrosion of chromium-containing dental alloys. Chin. Chem. Lett. 31 (1), 145–149. 10.1016/j.cclet.2019.07.026

[B25] Yang X. Wang B. Sha D. Liu Y. Liu Z. Shi K. (2021). PVA/Poly(hexamethylene guanidine)/gallic acid composite hydrogel films and their antibacterial performance. ACS Appl. Polym. Mater 3 (8), 3867–3877. 10.1021/acsapm.1c00447

[B26] Yuan H. Xue C. Zhu J. Yang Z. Lan M. (2021). Preparation and antifouling property of polyurethane film modified by PHMG and HA using layer-by-layer assembly. Polym. (Basel) 13 (6), 934. 10.3390/polym13060934 PMC8002859 33803560

[B27] Zhang J. Luo H. Yin X. Shi Y. Zhang Y. Tan L. (2021). Surface coating on aluminum substrate with polymeric guanidine derivative to protect jet fuel tanks from microbial contamination. Surf. Coat. Technol. 422, 127521. 10.1016/j.surfcoat.2021.127521

[B28] Zhang M. Liu X. Xie Y. Zhang Q. Zhang W. Jiang X. (2020). Biological safe gold nanoparticle-modified dental aligner prevents the porphyromonas gingivalis biofilm formation. ACS Omega 5 (30), 18685–18692. 10.1021/acsomega.0c01532 32775870 PMC7407536

[B29] Zhang N. Bai Y. Ding X. Zhang Y. (2011). Preparation and characterization of thermoplastic materials for invisible orthodontics. Dent. Mater J. 30 (6), 954–959. 10.4012/dmj.2011-120 22123023

[B30] Zhao W. Kumar Kundu C. Li Z. Li X. Zhang Z. (2021). Flame retardant treatments for polypropylene: Strategies and recent advances. Compos. Part A Appl. Sci. Manuf. 145, 106382. 10.1016/j.compositesa.2021.106382

[B31] Zheng L. Li S. Luo J. Wang X. (2020). Latest advances on bacterial cellulose-based antibacterial materials as wound dressings. Front. Bioeng. Biotechnol. 8, 593768. 10.3389/fbioe.2020.593768 33330424 PMC7732461

[B32] Zhong X. Li R. Wang Z. Wang Y. Wang W. Yu D. (2021). Highly flexible, transparent film prepared by upcycle of wasted jute fabrics with functional properties. Process Saf. Environ. Prot. 146, 718–725. 10.1016/j.psep.2020.12.013

[B33] Zhou Y. Jiang Y. Zhang Y. Tan L. (2022). Improvement of antibacterial and antifouling properties of a cellulose acetate membrane by surface grafting quaternary ammonium salt. ACS Appl. Mater. Interfaces 14 (33), 38358–38369. 10.1021/acsami.2c09963 35950600

