# Supplementary material for: Development of an antibacterial polypropylene/polyurethane composite membrane for invisible orthodontics application
Source: Front Bioeng Biotechnol. 2023 Jul 7;11:1233398. doi: 10.3389/fbioe.2023.1233398 (PMC10361250; doi:10.3389/fbioe.2023.1233398)
Supplement: Supplementary file 1 [file DataSheet1.docx]

Supporting Information

**Development of an antibacterial polypropylene/polyurethane composite membrane for invisible orthodontics application**

Feng Yang^1,2,3^, Chenyi Wu^1^, Yuanzhang Jiang^2,3^, Lin Tan^2,3^, Rui Shu^1*^

^1^Department of Pediatric Dentistry, West China School of Stomatology, State Key Laboratory of Oral, Diseases, Sichuan University, Chengdu, 610041 China;

^2^College of Biomass Science and Engineering, State Key Laboratory of Polymer Materials Engineering, Sichuan University, Chengdu 610065, China;

^3^Yibin Institute of Industrial Technology/Sichuan University, Research Center for Fiber Science and Engineering Technology, Yibin Park, Yibin 64460, China.

*Corresponding to: Dr./Ass. Prof. Rui Shu (E-mail:* [*shurui@scu.edu.cn*](mailto:shurui@scu.edu.cn)*)*

1. Synthesis route of PHMG-SS

Figure S1. Synthesis roadmap of PHMG-SS.


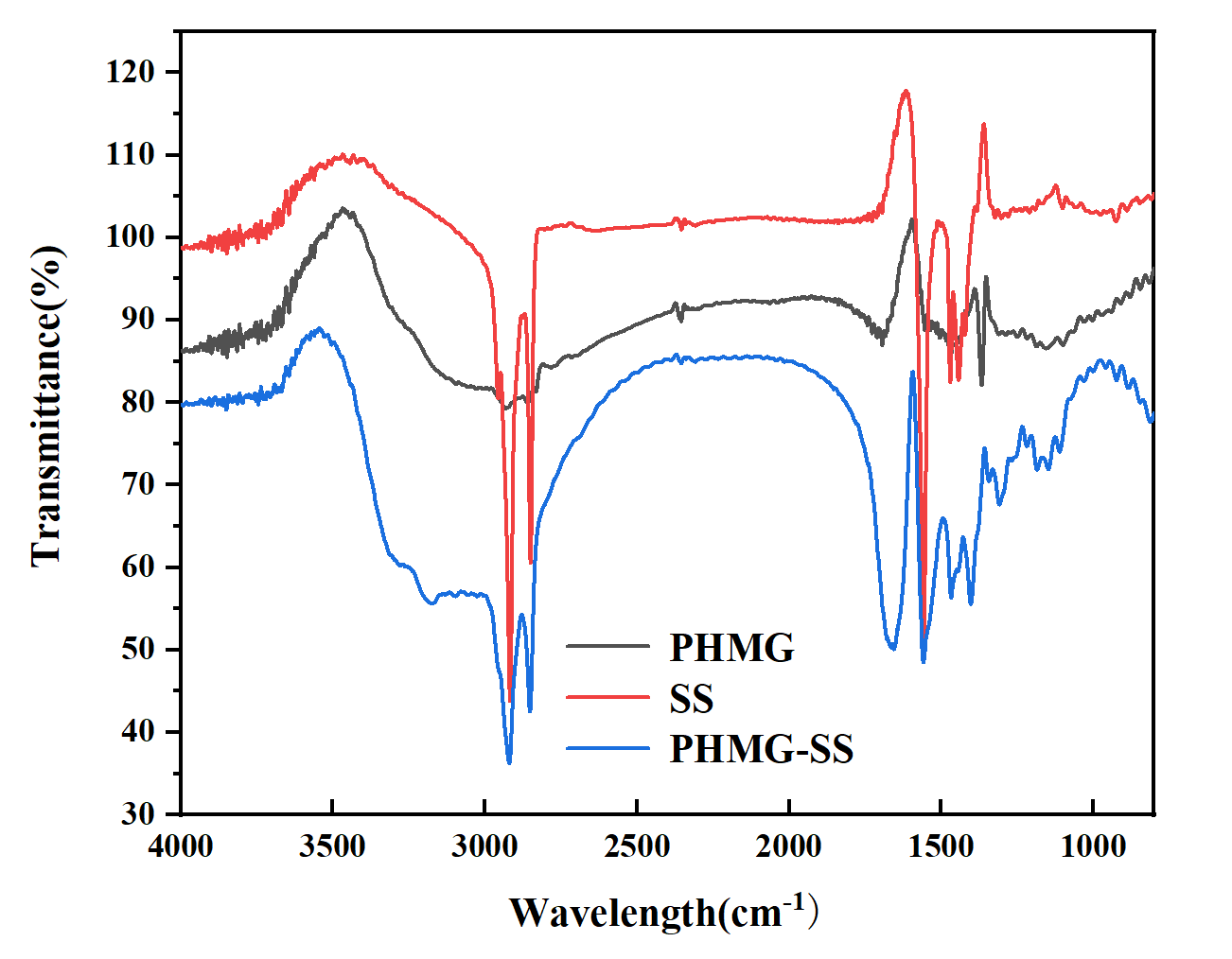


Fig. S2. FITR curves of PHMG, SS and PHMG-SS.

Fig. S2 demonstrated the FITR spectrum of SS, PHMG, and PHMG-SS. The absorption peak at 1600 cm^–1^ can be ascribed to the stretching vibration of C-N and N-H, which can be observed in FTIR curves of PHMG and PHMG-SS. Meanwhile, the characteristic peak at 1555 cm^–1^ can be attributed to the stretching vibration absorption peak of -COO- of SS, and PHMG-SS also shows the same characteristic peak. These characteristic peaks indicate the successful synthesis of PHMG-SS.


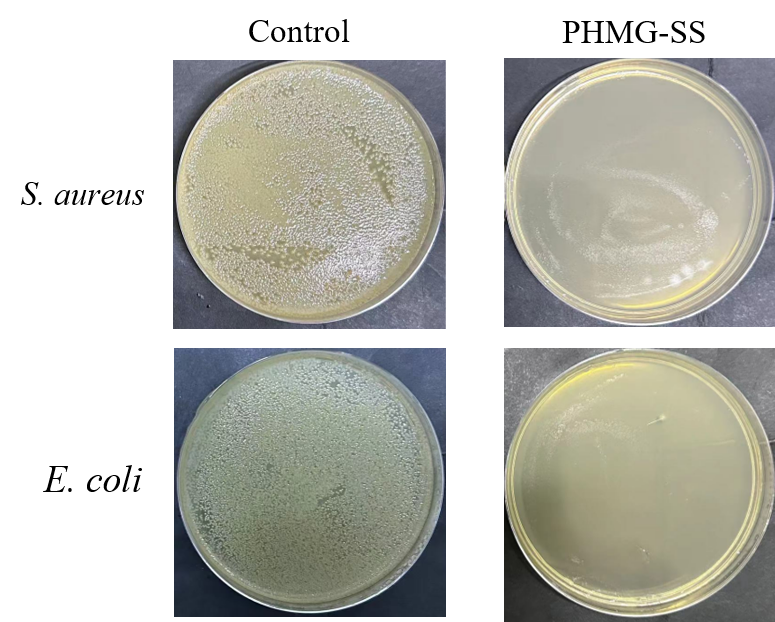


Fig. S3. The antibacterial properties of the synthetic PHMG-SS

The antibacterial performance of PHMG-SS was shown in Fig. S3, indicating its excellent antibacterial properties against *S. aureus* and *E. coli*.

Table S1. Moisture measurements of PP and TPU before and after drying.

|  | PP（ppm） | TPU（ppm） |
| --- | --- | --- |
| Before drying  After drying | 90  83 | 2585  277 |


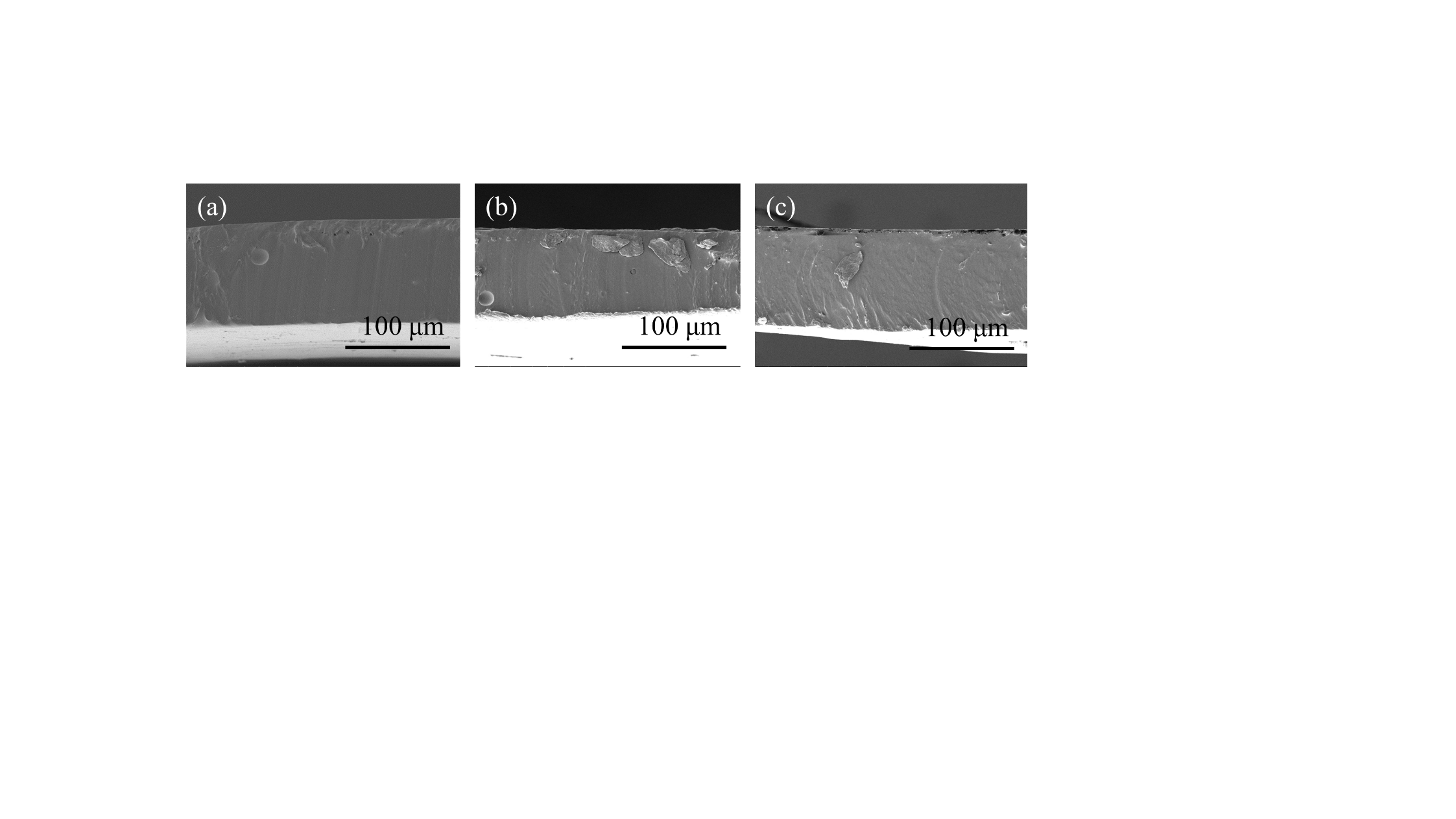
Fig. S4. SEM images of the cross-section of P4T6 (a), P4T6 (0.5%) (b), and P4T6 (1%) (c)


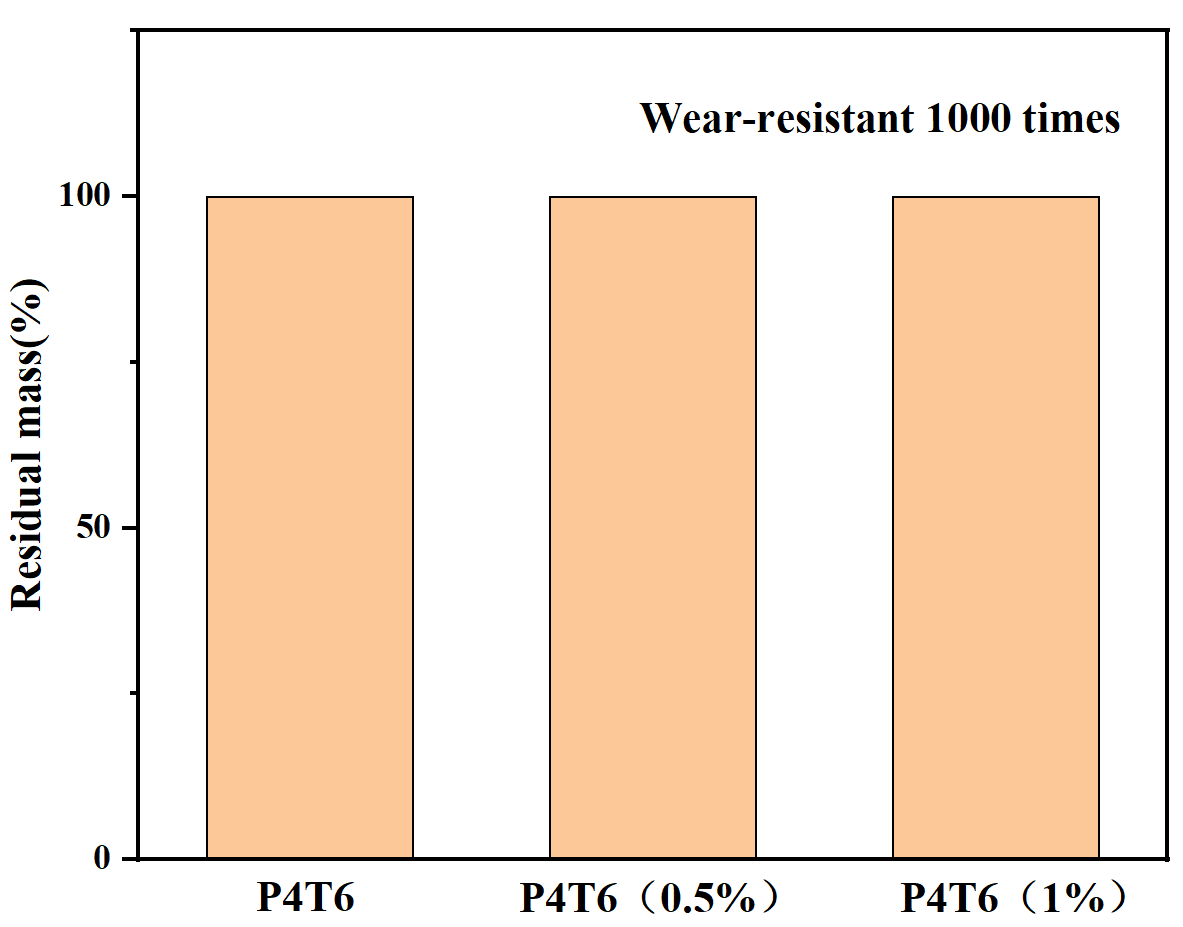


Fig. S5. Abrasion resistance tests.


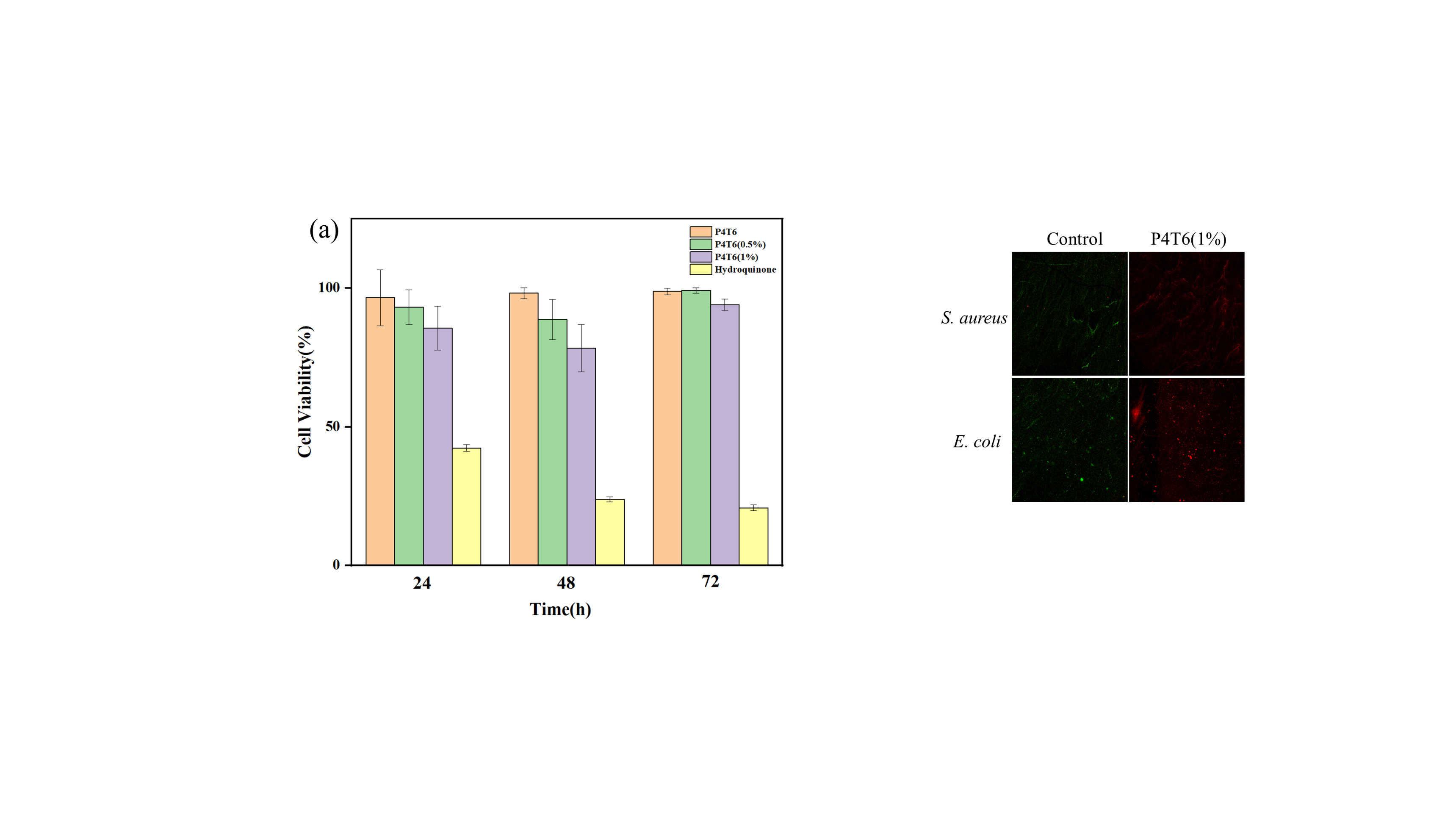


Fig. S6. CLSM images of *S. aureus* and *E. coli* on TCMs.

The fluorescent staining of live & dead bacteria was also shown in the Fig. S7. In the control group P4T6, *S. aureus* and *E. coli* (green fluorescence) can be found, while the experimental group P4T6 (1%) basically exhibits no green fluorescence, and all show red fluorescence, which means that almost all bacteria (*S. aureus*, *E. coli*) are killed.


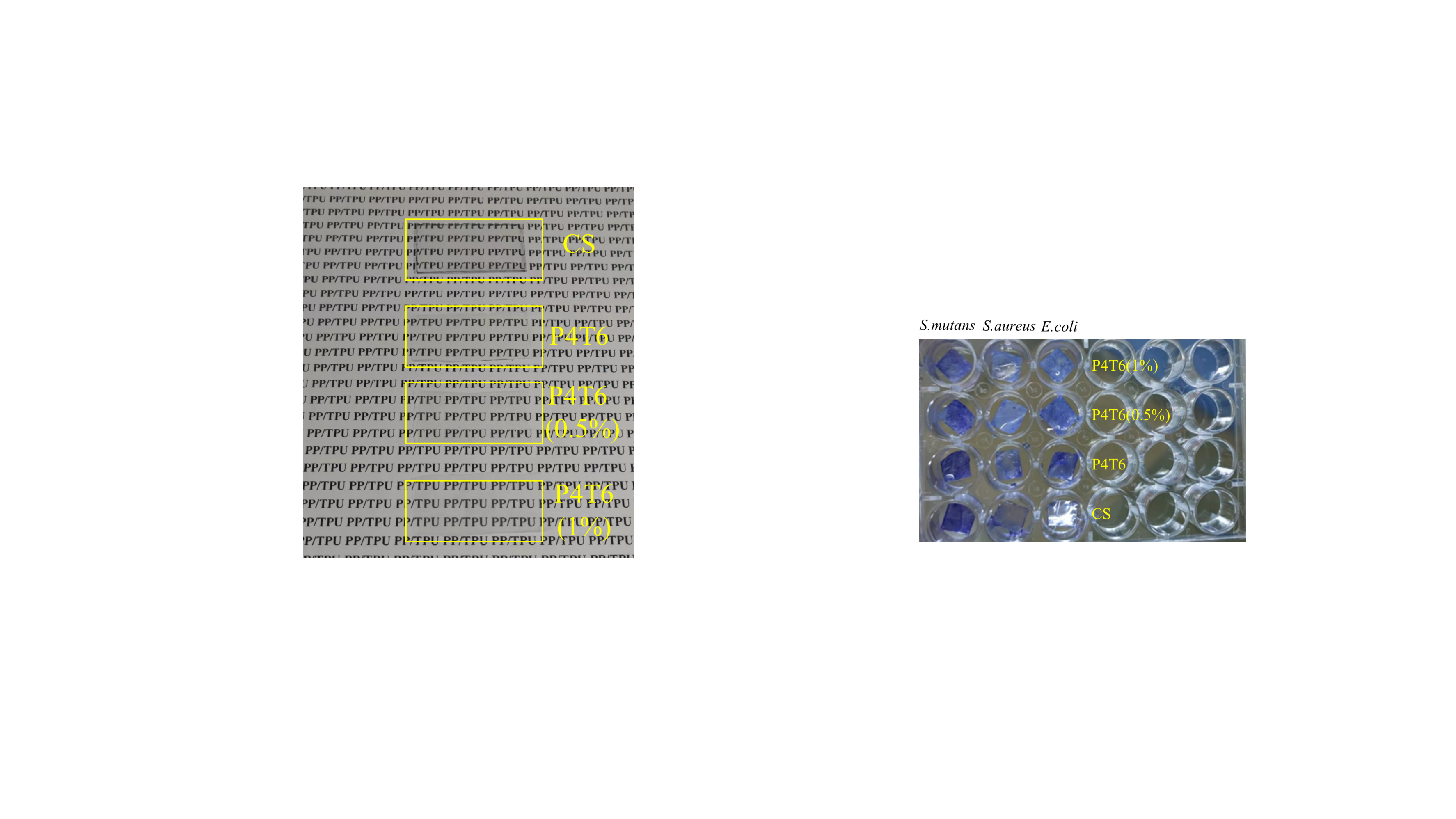


Fig. S7. The photo of biofilms stained by crystal violet.
